# Supplementary material for: Light intensity drives different growth strategies in two duckweed species: Lemna minor L. and Spirodela polyrhiza (L.) Schleiden
Source: PeerJ. 2021 Dec 20;9:e12698. doi: 10.7717/peerj.12698 (PMC8697765; doi:10.7717/peerj.12698)
Supplement: Supplemental Information 1 — Statistically significant effects in MANOVA after the Box–Cox transformation are marked in bold (for MANOVA results see Table 1); three light intensities were applied: low–125, medium–236 and high–459 µmol photons m–2 s–1. [file peerj-09-12698-s001.docx]

| Effects | Factors | | | N | rN | | rS | |
| --- | --- | --- | --- | --- | --- | --- | --- | --- |
|  |  |  |  |  | Mean | SD | Mean | SD |
| Total |  |  |  | 120 | 0.21 | 0.025 | 0.24 | 0.030 |
| **Light intensity** | low |  |  | 40 | 0.20 | 0.023 | 0.23 | 0.029 |
|  | medium |  |  | 40 | 0.22 | 0.022 | 0.25 | 0.029 |
|  | high |  |  | 40 | 0.20 | 0.028 | 0.23 | 0.032 |
| **Habitat** | shady |  |  | 60 | 0.19 | 0.022 | 0.22 | 0.022 |
|  | open |  |  | 60 | 0.22 | 0.020 | 0.26 | 0.018 |
| **Species** | *L*. *minor* |  |  | 60 | 0.21 | 0.027 | 0.25 | 0.030 |
|  | *S*. *polyrhiza* |  |  | 60 | 0.20 | 0.020 | 0.23 | 0.027 |
| Light intensity * habitat | low | shady |  | 20 | 0.18 | 0.020 | 0.21 | 0.020 |
|  | low | open |  | 20 | 0.22 | 0.012 | 0.26 | 0.014 |
|  | medium | shady |  | 20 | 0.20 | 0.015 | 0.22 | 0.018 |
|  | medium | open |  | 20 | 0.23 | 0.017 | 0.27 | 0.016 |
|  | high | shady |  | 20 | 0.19 | 0.026 | 0.21 | 0.024 |
|  | high | open |  | 20 | 0.21 | 0.025 | 0.26 | 0.020 |
| **Light intensity * species** | low | *L*. *minor* |  | 20 | 0.20 | 0.030 | 0.24 | 0.033 |
|  | low | *S*. *polyrhiza* |  | 20 | 0.20 | 0.011 | 0.23 | 0.023 |
|  | medium | *L*. *minor* |  | 20 | 0.22 | 0.025 | 0.26 | 0.028 |
|  | medium | *S*. *polyrhiza* |  | 20 | 0.21 | 0.015 | 0.24 | 0.027 |
|  | high | *L*. *minor* |  | 20 | 0.22 | 0.020 | 0.25 | 0.026 |
|  | high | *S*. *polyrhiza* |  | 20 | 0.18 | 0.021 | 0.22 | 0.029 |
| **Habitat * species** | shady | *L*. *minor* |  | 30 | 0.19 | 0.023 | 0.23 | 0.022 |
|  | shady | *S*. *polyrhiza* |  | 30 | 0.19 | 0.020 | 0.21 | 0.017 |
|  | open | *L*. *minor* |  | 30 | 0.23 | 0.011 | 0.27 | 0.010 |
|  | open | *S*. *polyrhiza* |  | 30 | 0.20 | 0.016 | 0.25 | 0.015 |
| Light intensity * habitat * species | low | shady | *L*. *minor* | 10 | 0.18 | 0.025 | 0.22 | 0.028 |
|  | low | shady | *S*. *polyrhiza* | 10 | 0.19 | 0.007 | 0.21 | 0.006 |
|  | low | open | *L*. *minor* | 10 | 0.22 | 0.005 | 0.27 | 0.006 |
|  | low | open | *S*. *polyrhiza* | 10 | 0.21 | 0.011 | 0.25 | 0.011 |
|  | medium | shady | *L*. *minor* | 10 | 0.20 | 0.018 | 0.24 | 0.019 |
|  | medium | shady | *S*. *polyrhiza* | 10 | 0.20 | 0.010 | 0.21 | 0.009 |
|  | medium | open | *L*. *minor* | 10 | 0.24 | 0.010 | 0.28 | 0.012 |
|  | medium | open | *S*. *polyrhiza* | 10 | 0.22 | 0.013 | 0.26 | 0.014 |
|  | high | shady | *L*. *minor* | 10 | 0.20 | 0.013 | 0.23 | 0.012 |
|  | high | shady | *S*. *polyrhiza* | 10 | 0.17 | 0.027 | 0.20 | 0.025 |
|  | high | open | *L*. *minor* | 10 | 0.24 | 0.006 | 0.27 | 0.008 |
|  | high | open | *S*. *polyrhiza* | 10 | 0.19 | 0.008 | 0.24 | 0.011 |
